# Porous organic cages stabilize methylammonium lead iodide films

**DOI:** 10.1038/s42004-022-00781-6

**Published:** 2022-11-24

**Authors:** Victoria Richards

**Affiliations:** Communications Chemistry, https://www.nature.com/commschem/

**Keywords:** Photovoltaics, Porous materials, Coordination chemistry

## Abstract

Organic–inorganic lead halide perovskites are highly promising materials for solar cell devices, but their widespread commercialization is hindered by their poor environmental stability. Now, porous organic cages are shown to stabilize methylammonium lead iodide films under hot and humid conditions.

Solar cells based on organic–inorganic lead halide perovskites are capable of achieving outstanding solar-to-electricity conversion efficiencies, but these materials degrade rapidly in the presence of heat, light, oxygen or moisture. Efforts to improve perovskite stability have so far centered on introducing additives to their synthesis, such as salts, small molecules, polymers or nanoparticles, with an underlying focus on improving material crystallinity and passivating defects in polycrystalline films. Now, an international collaboration led by Shijing Sun and Tonio Buonassisi at MIT in the US and Ming Liu and Andy Cooper at the University of Liverpool in the UK demonstrate that porous organic cages can significantly stabilize methylammonium lead iodide (MAPbI_3_) films, even though the cages induce disorder rather than ordered grain growth [10.1021/acs.chemmater.2c01502]^1^.

The team posited that the adsorption properties of porous organic cages could allow them to act as a sort of desiccant, mopping up unwanted molecules or ions that cause MAPbI_3_ degradation. Thanks to its low-temperature solution processability, the researchers easily combined RCC3—a widely studied molecular cage possessing 12 amine groups within its cavity—with perovskite precursors to produce perovskite–cage thin films. Their degradation behaviour was then tested in a custom-built environmental chamber at MIT, where an in situ optical setup quantifies the onset of degradation in real time. “These cage molecules surprised us”, comments first author Shijing Sun. “I remember a few times where my labmates and I asked each other “Why aren’t these perovskite–cage films degrading?!” We were planning to use the environmental chamber for other projects this week!”, she jokes. Indeed, the team witnessed an up to 50x delay in the onset of degradation in comparison to MAPbI_3_ films, with the MAPbI_3_–RCC3 composites remaining structurally and chemically stable for several days at 85 °C and 85% relative humidity.

**Figure:**
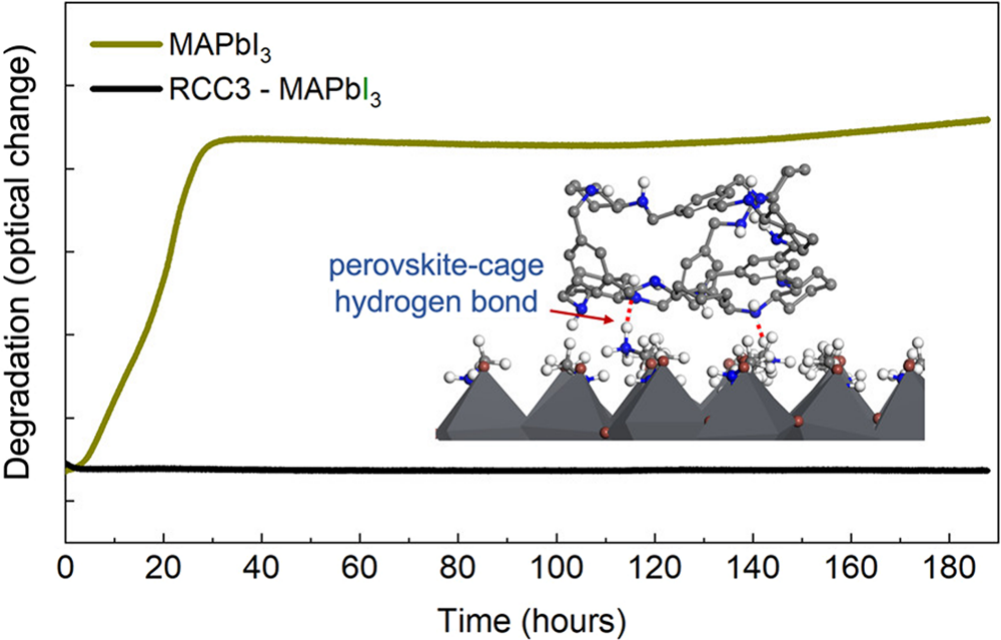
AMERICAN CHEMICAL SOCIETY

To understand why, the team used a combination of attenuated total reflection Fourier transform infrared spectroscopy and ab initio molecular dynamics simulations to study the stabilization mechanism. They found that the amine groups within the cavities and on the surfaces of the RCC3 cages form strong hydrogen bonding interactions with methylammonium (MA^+^) cations—a volatile molecule whose dissolution is known to be one of the dominant mechanisms that contributes to MAPbI_3_ degradation. The cages distributed within the bulk were postulated to inhibit the MA^+^ ions from escaping, while an amorphous RCC3 network on MAPbI_3_ is suggested to act like a sticky layer that suppresses MA^+^ motion.

The researchers performed proof-of-concept testing that confirms enhanced reliability within photovoltaic and light-emitting devices, but conclude that future device optimization is required to navigate performance–stability trade-offs. The team also hope that their study will inspire others to explore perovskite–cage composite films. “Moving forward, we believe that there are many candidates in both the porous and dense materials families to be explored, with porous organic cages providing a rich platform for designing and optimizing additives to stabilize perovskite thin films in the future.”, concludes Sun.
